# Visual Search in Ecological and Non-Ecological Displays: Evidence for a Non-Monotonic Effect of Complexity on Performance

**DOI:** 10.1371/journal.pone.0053420

**Published:** 2013-01-08

**Authors:** Philippe Chassy, Fernand Gobet

**Affiliations:** 1 Department of Psychology, Liverpool Hope University, Liverpool, United Kingdom; 2 Centre for the Study of Expertise, Brunel University, Uxbridge, United Kingdom; Monash University, Australia

## Abstract

Considerable research has been carried out on visual search, with single or multiple targets. However, most studies have used artificial stimuli with low ecological validity. In addition, little is known about the effects of target complexity and expertise in visual search. Here, we investigate visual search in three conditions of complexity (detecting a king, detecting a check, and detecting a checkmate) with chess players of two levels of expertise (novices and club players). Results show that the influence of target complexity depends on level of structure of the visual display. Different functional relationships were found between artificial (random chess positions) and ecologically valid (game positions) stimuli: With artificial, but not with ecologically valid stimuli, a “pop out” effect was present when a target was visually more complex than distractors but could be captured by a memory chunk. This suggests that caution should be exercised when generalising from experiments using artificial stimuli with low ecological validity to real-life stimuli.

## Introduction

Under some circumstances, the ability to detect a target makes the difference between life and death (e.g., detecting cars at a crossroads). Considerable research has been carried out in cognitive psychology and cognitive neuroscience on visual search behaviour, focussing on questions such as the number of targets, target-distractor discriminability, distractor complexity, and whether information is processed sequentially [1]. However, most of this research has been carried out with artificial stimuli and little is known about how search for complex targets is performed in ecologically valid environments – that is in experimental environments that approximate the characteristics of the real-life situation under study (Neisser, 1976). (Note that these environments can be natural or human-made. What matters is that the experimental situation relates to a situation with which participants are intimately familiar. Thus, both trekking in the wilderness and crossing a road could be the source of ecologically valid experiments.) In the few studies using ecologically valid stimuli, the exploration of the mechanisms underpinning human visual search has revealed that experts can detect domain-specific patterns faster than novices [2]. This finding has led psychologists to propose that domain-specific knowledge directs attention towards potentially relevant locations [3]. Strikingly, sophisticated models of attention (e.g., [1]) account for a wide range of findings but do not address domain-specific guidance of attention in ecologically valid situations. The high importance of complex targets in ecologically valid settings [4], on the one hand, and the established influence of knowledge in exploring visual scenes [5], [6] on the other hand, call for research remedying this gap.

Complexity is a concept that displays variations in its definition. A traditional definition is that of a system made of numerous components which interact. Due to the actual difficulty in analysing systems at the level of units, some researchers have approached complexity by analysing how the system as a whole behaves. Complexity is then often defined by the potential states that the system can display (e.g., cyclical, chaotic, or self-organising). Considered in the context of cognitive psychology, and particularly when referring to perceptual processes, complexity refers to the visual or auditory properties of a stimulus. Close to the traditional definition, complexity in the present article refers to the combination of units (i.e., pieces) and interactions (attack or defence dynamics linking the pieces).

One reason for the lack of research on the role of complexity and expertise in ecologically valid tasks is the difficulty of finding an appropriate environment. The environment used must have several crucial characteristics: it should be possible to present situations visually for a brief period of time; stimulus complexity should be both controllable by the experimenter and ecologically valid; and there should be a way to measure complexity. Finally, given the difficulty of measuring expertise in most domains [7], the environment should ideally have an internal and ecologically valid measure of skill. Chess is a unique environment, in that it meets all these criteria. In particular, it offers a controllable and ecologically valid measure of complexity: the number of occupied and empty squares implicated in a particular pattern. Since chess is an environment designed by humans, it also minimizes the influence of hard-wired search strategies designed by evolution to explore visual scenes [8]. This makes chess a good candidate for exploring the influence of knowledge on visual search. This paper aims to close this gap in the literature by examining the extent to which domain-specific knowledge facilitates the processing of scenes of varying visual complexity. It does this by contrasting the performance in ecologically valid and ecologically invalid settings.

Chase and Simon [9] posited that experts’ perceptual advantage was due to them having chunks of visual information (typical piece configurations on the board) stored in long-term memory. Upon recognition of a chunk, attention is directed towards potentially salient locations and potential moves. The studies conducted with chess have confirmed that domain-specific knowledge orients attention in ecological stimuli, making experts faster than novices in detecting targets [2], [10]. Since the amount of information captured by perception at any moment is limited [11], and the understanding of a problem situation takes several eye fixations [3], [12], an important effect of complexity on cognition is to increase the cost of processing information [10]. Whether this effect can be attenuated by expertise is as yet unknown.

Chess has long been a central paradigm for understanding the components of expertise [13]. However, the studies carried out to study the influence of knowledge (expertise) on complexity in search tasks [2], [14]–[18] suffer from several infelicities in their design that make comparison between studies difficult and thus limit the general conclusions that can be drawn. These include: small samples (e.g., three participants in a group; [2]), differences in board size (e.g., 8×8 vs. 3×3 squares) differences in target salience (minor piece [bishop or knight] vs. king), unsystematic manipulation of complexity and different definitions of skill levels across experiments. In the present study, we addressed these issues by systematically manipulating complexity and recruiting participants spanning a wide range of expertise levels.

Based on dominant theories of perceptual expertise, such as chunking [9] and template theories [19], we made several predictions. First, since complexity increases the amount of information to process, we expected an effect of complexity on both performance and processing time. Second, considering that domain-specific knowledge orients attention to relevant locations in structured but not in unstructured scenes, unstructured stimuli should force the perceiver to develop new strategies to explore the visual scene. Thus, and in line with previous findings, the time taken to find a target should be longer in unstructured stimuli than in structured stimuli. Third, considering that the knowledge of the perceiver is used to structure the stimuli and that a lack of structure will lead to a perceptual overload, we expected complexity to interact with structure. In particular, we examined the effect of complexity on ecologically valid stimuli. This prediction relates to the hypothesis that chunking makes it possible to recode and compress information, and thus to reduce perceptual complexity.

## Methods

### Participants

Twenty-nine chess players (1 female) were recruited from several chess clubs in France and the Netherlands. All participants had normal or corrected-to-normal vision. The mean rating was *M = *1681 Elo (*SD = *262.69 Elo) and the ratings ranged from 1240 to 2300. (The Elo rating is a scale widely used in the chess world to measure chess skill; see Elo [20] for details.) Two levels of expertise were defined by splitting players around the median of Elo rating (median = 1622 Elo). The novice group comprised 14 players (*M* = 1470 Elo; *SD* = 101 Elo). The club players group consisted of 15 players (*M* = 1907 Elo; *SD* = 176 Elo). The difference between the means of the two groups (437 Elo) reflects a huge difference in skill *t*(27) = −8.28, *p*<.001, which translates into a probability of winning for the best player equal to *p* = .94.

### Procedure

Participants were instructed that the purpose of the study was to understand how chess players carry out king, check and checkmate detections. After signing a consent form, they were asked whether they had a past of epileptic seizure (i.e., evidence of photosensitivity) and whether they were familiar with chess programs. None of the participants were photosensitive and all were familiar with chess programs. Participants sat 40 cm from the screen of a Toshiba laptop. The experiment was conducted individually in a quiet and well-lit room. After completion of the experiment, they received €10.

Participants were asked to go through a detection task that implemented three levels of visuospatial pattern complexity (henceforth, complexity): detection of white king, detection of check, and detection of checkmate. The targets were present in half of the trials, and absent in the other half. Structure was manipulated by using either ecologically valid stimuli (i.e., game positions) or ecologically invalid stimuli (i.e., random positions). Participants had to complete 40 king detections, 40 check detections, and 40 checkmate detections. There were two orders, which were counterbalanced across the participants: (a) king, check, and checkmate, and (b) checkmate, check, and king. In each trial, participants’ response, latency, and accuracy were recorded.

As in the previous paragraphs, the term *complexity* refers to the complexity of the stimulus. In spite of the change in instructions, the task remains the same: to detect a visual pattern. It must be noted that from chess players’ point of view, a chess position contains many patterns with two or more pieces, a same piece being able to belong to different patterns. Some patterns (e.g., *fianchetto*) present little variability and are predictable in terms of their location over the board and amount of pieces. We needed patterns that can occur in different places of the board and that can vary in complexity. The check and checkmate situations meet these criteria. The chosen approach allowed us both to provide the participants with ecological instructions and to present the experimenters with a new, valid method for measuring complexityMaterial.

For each level of complexity (i.e., king, check, checkmate), ten game positions were selected randomly from a database of 2,000,000 games. To ensure a good ecological validity, only games from experts were used. Complexity was measured as the total number of squares (both occupied squares [pieces] and unoccupied squares) implicated in the target pattern. For the king condition, one piece (the king) and zero empty squares had to be detected, thus complexity equalled 1 square. For the check condition, the patterns were made of 2 pieces (*SD = *0 pieces) and 1.9 empty squares (*SD = *1.73), on average. Hence, the average space to be covered to recognise the pattern was of 3.9 squares on average (*SD = *1.73 squares). Finally, for the checkmate conditions, the patterns were made of 4.7 pieces (*SD = *1.06 pieces) and 17.7 empty squares (*SD = *2.45 squares). Hence, the space to be covered to recognise the checkmate patterns was of 22.4 squares on average (*SD = *3.03 squares). In summary, the average complexity of patterns spanned three levels: 1 square (king), 3.9 squares (check), and 22.4 squares (checkmate). As the number of pieces on the board was similar in the three conditions (*M* = 23.5 pieces, *SD* = 4.06 pieces), the visual load was controlled for.

**Figure 1:**
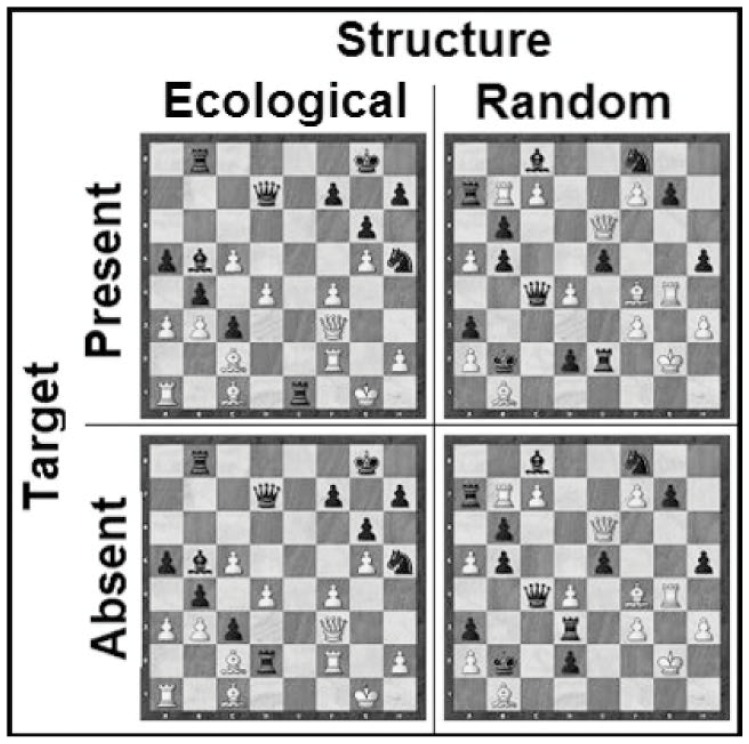
Stimuli sample for the check condition.

The game positions were used to generate non-target and random stimuli. Non-target stimuli were generated either by deleting the white king (king detection) or by moving the attacking piece to a new, non-attacking square (check and checkmate detection). Note that it was necessary to keep the king on the board in the check and checkmate conditions, because getting rid of the king would make the non-target conditions similar to no-king condition. By applying these procedures, we obtained ten more positions per level of complexity. Finally, random positions were created by randomly reallocating the pieces of each position to a new square. A constraint was that the white king could only end up on a square occupied by a white king in one of the nine other positions. Targets were present in half the random positions and absent in the other half. Figure 1 presents an example of a stimulus in each experimental condition for the check level of complexity.

## Results

The presentation of results is divided in three sections. In the first section we outline the procedure used to pre-process the data. The second section presents the results for all positions and reports whether complexity and structure affected players’ response time and proportion of correct answers. In the third section, we focus on ecologically valid situations.

### Data Trimming and Transformation of Response Times

To ensure that the inferential tests were carried out without violation of the statistical assumptions, we used a systematic procedure for the response times (RTs). For each participant, the first step was to trim the data by discarding trials with an RT less than 200 ms or superior to the mean plus three standard deviations (3.26% of observations). The second step was to discard all trials in which the participants did not answer correctly (8.61% of observations after trimming). The third step was to test whether the data were normally distributed. Since the Kolmogorov-Smirnov revealed that the normality assumption could not be held, all RTs were log-transformed. Whenever Mauchly’s test indicated that the distribution of data was not spherical, the most conservative correction (i.e., half bound) was used to adjust the F values of the ANOVA.

### General Results

The means and standard deviations for each experimental condition are reported in Table 1. The upper half of the table reports the RTs, and the lower half reports the proportion of correct responses. Overall, the participants performed the detection task in 3.96 s on average (*SD* = .82 s) and with high accuracy (*M* = .91, *SD* = .05).

**Table 1:** Reaction times (top panel) and proportion correct (bottom panel) as a function of complexity, structure, target presence, and skill level.

			King				Complexity Check				Checkmate			
			Structure											
			Random		Game		Random		Game		Random		Game	
			Target											
			A	P	A	P	A	P	A	P	A	P	A	P
	N	M	2.16	1.53	1.83	1.21	3.70	2.20	3.27	2.12	6.89	7.90	6.60	8.04
RT		SD	0.42	0.48	0.44	0.23	0.85	0.67	0.92	0.54	2.09	1.81	1.71	1.90
	C	M	2.06	1.44	1.79	1.26	3.29	1.90	2.94	1.70	6.61	7.73	6.24	7.59
		SD	0.52	0.38	0.56	0.32	0.96	0.59	1.05	0.46	3.03	3.59	2.84	4.20
	N	M	0.99	0.82	0.99	0.93	0.96	0.94	0.89	0.93	0.86	0.83	0.91	0.90
Prop.		SD	0.03	0.16	0.04	0.07	0.06	0.08	0.10	0.11	0.11	0.15	0.11	0.12
	C	M	0.97	0.91	0.99	0.99	0.95	0.94	0.91	0.99	0.91	0.93	0.93	0.94
		SD	0.08	0.11	0.03	0.04	0.11	0.08	0.05	0.03	0.09	0.07	0.08	0.06

Note. RT: response time, Prop.: Proportion of correct responses, N: Novices, C: Club players A: Absent, P: Present.

We carried out an analysis of variance on RT and proportion correct separately, using a 2 (expertise) × 2 (structure) × 2 (target presence) × 3 (complexity) mixed design. Expectedly, there was a main effect of expertise on performance *F*(1, 27) = 5.01, *p*<.05, MSE = 0.02. However, the two expertise groups did not differ in RTs, *F*(1, 27) = .94, *p* = .34, MSE = 0.15. Consistent with previous research, we found a significant main effect of structure on RT, *F*(1, 27) = 91.97, *p*<.01, MSE <0.01, and performance, *F*(1,27) = 8.96, *p*<.01, MSE <0.01. Participants were slower in completing the task with random positions (*M*3.96 s; *SD* = 3.01 s) than with game positions (*M* = 3.72 s; SD = 2.93 s); and performance with game positions (*M* = .94, *SD* = 0.08) was higher by 2% than with random positions (*M* = .92, *SD* = .11). Target presence (*M* = 3.72 s; *SD* = 2.47 s) was on average spotted faster than target absence (*M* = 3.95 s; *SD* = 3.40 s), *F*(1, 27) = 272.46, *p*<.01, MSE = 0.02. Yet target had no effect on performance *F*(1, 27) = 2.51, *p = *.12, MSE = 0.01. Crucially, complexity significantly affected RTs and performance, *F*(2, 54) = 609.22, *p*<.01, MSE = 0.02 and *F*(2, 54) = 10.27, *p*<.01, MSE <0.01, respectively. With increasing levels of complexity, the mean RT increased (*king M* = 1.66 s, *SD* = .53 s; *check M* = 2.65 s, *SD* = 1.03 s; and *checkmate M = *7.21 s, *SD* = 2.75 s) and the mean performance decreased (*king M* = .95, *SD* = .10; *check M* = .94, *SD* = .08, *checkmate M* = .90, *SD = *.11). Note that the effect is considerable with RTs: a factor of 1.7 between the king and check conditions, and a factor of 4.3 between the king and checkmate conditions.

**Figure 2:**
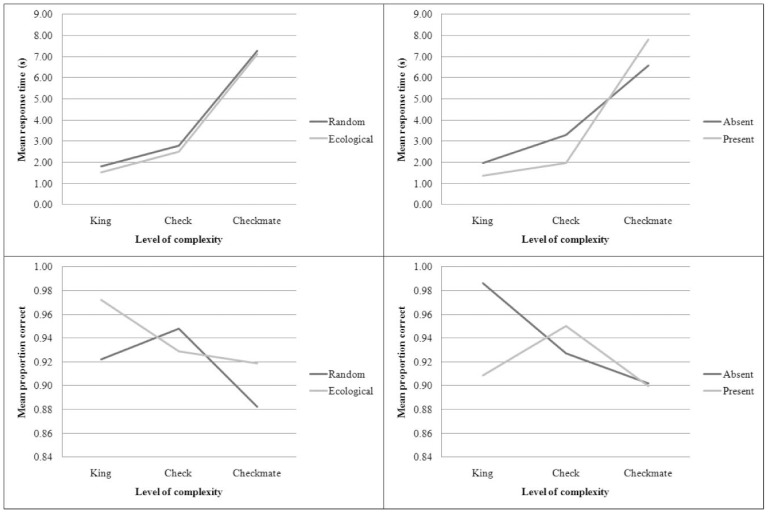
Interactions between complexity and other factors.

Complexity interacted significantly with structure both with respect to RTs, *F*(2, 54) = 10.25, *p*<.01, MSE <0.01 and proportion correct, *F*(2, 54) = 8.90, *p*<.01, MSE <0.01. Complexity had an effect on the speed with which targets were detected, *F*(2, 54) = 132.57, *p*<.01, MSE <0.01 and performance, *F*(2, 54) = 10.28, *p*<.01, MSE <0.01. The significant interactions, which include complexity, are displayed in Figure 2. These interactions are very informative with respect to how complexity modulates the effects of structure and target. We first consider the interaction between complexity and structure, starting with RTs. In random stimuli, when complexity increased, the participants tended to reduce their response criterion compared to ecologically valid stimuli as shown by the fact that the curves converge. With performance, the pattern is much different. With game positions, the increase in complexity was reflected by a decrease in performance. With random positions, however, mean performance reached a peak in the intermediate level of complexity and then dropped dramatically. This finding is also reflected by the interaction between complexity and target presence: In spite of using much more time in the highest level of complexity, the performance dropped dramatically.

Visual inspection of the third quadrant of Figure 2 suggests that, while proportion correct is a linear function of level of complexity for the game positions, it is a quadratic function for the random stimuli. It could be argued that there is a speed-accuracy trade-off in our results, which would make it hard to interpret them. However, Figure 2 also shows that, overall, while response time increases from the King to the Checkmate condition, accuracy *decreases*. This is the opposite pattern to what would be observed if there was a speed-accuracy trade-off. Statistical analysis supported this impression. While trend analysis found only a statistically significant linear term for the game condition, *F*(1, 28) = 24.51, *p*<.01 it found both a linear and a quadratic term for the random condition, *F*(1, 28) = 7.25, *p*<.05, and *F*(1, 28) = 9.24, *p*<.01, respectively. These crucial results show that performance in random stimuli, but not in game stimuli, follow a curvilinear progression. The influence of complexity on random stimuli can be investigated further by modelling it by a quadratic equation. Equation 1 shows the relationship between complexity (C) and proportion of correct hits (P).



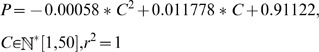
(1)

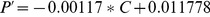
(2)


Interestingly, this equation predicts that performance will reach zero when complexity is equal to 51 squares. Hence, there is a maximal complexity that can be handled by memory. In addition, the analysis of the derivative (see Equation 2) indicates that performance peaks when C = 10.1, i.e. when the complexity of the target pattern covers 10 squares. Hence, 10 squares is where the pop out effect peaks, making performance close to perfection (P = .97).

### Focus on Ecologically Valid Stimuli

When the analysis with expertise and complexity as independent variables, and RTs and performance as dependent variables, was restricted to ecologically valid conditions (i.e., game positions), an opposite pattern was found: Expertise significantly affected performance, *F*(1,27) = 6.42, *p*<.05, MSE = 0.01, but complexity failed to reach significance, *F*(1,27) = 3.02, *p = *.06, MSE <0.01. By contrast, complexity affected RT but expertise did not, *F*(1,27) = 555.88, *p*<.01, MSE = 0.02, *F*(1,27) = 1.39, *p = *0.25, MSE = .04, respectively.

Next, we tested the hypothesis that spatial dynamics are encapsulated in chunks. Under the assumption that empty squares are essential for attack-defence relationships, we predicted that empty space affects perceptual speed. When we regressed RT on complexity (C) (see Table 2), we found a nearly perfect linear relationship (see Equation 3), *F*(1, 2) = 1,021.15, *MSE* = 26.25, *p = *.02, r^2^ = .999. We found the same relationship when RTs were regressed on the number of empty squares (ES) (see Equation 4), *F*(1, 2) = 63,277, *MSE* = 26.27, *p<*.01, r^2^ = 1.
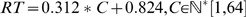
(3)

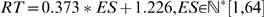
(4)


**Table 2:** Data used for regressions.

Level of Complexity	Complexity Components		Complexity	Dependent variable
	Piece	Empty square		RT
King	1	0	1	1.24
Check	2	1.9	3.9	1.92
Checkmate	4.7	17.7	22.4	7.83

The numbers are means.

Note. The data from novice and club players were pooled as no effect of expertise on RTs in ecologically valid situations was detected.

Equation 3 indicates that an increase in one complexity unit increases RT by 0.312 s, and Equation 4 shows that adding an empty square to the target pattern increases RT by an average of *M* = 0.373 s. The results strengthen the hypothesis that chunks encompass spatial information.

## Discussion

The present study investigated the effect of knowledge on recognition of visually complex patterns in a visual search task. To this purpose, novice and club chess players searched for visual targets of various complexity levels in random and game chess positions. We found main effects of expertise, structure and complexity on performance, as well as main effects of structure, target presence and complexity on response times. By large, these results replicate the well-established effects that expertise, structure, complexity and target presence have on behavioural indicators [2], [14]–[17], [21]. However, while previous research suggests that an increase in complexity entails an increase in difficulty, the present study has revealed an unexpected pattern of results: in unstructured environments, a medium level of complexity facilitates the detection of domain-specific targets. To the best of our knowledge, the present study is the first to show that complexity can facilitate perception in specific situations.

Crucially, the interaction between complexity and structure has uncovered a potential facilitator effect of complexity. With game positions, the proportion of correct responses is inversely proportional to complexity. However, with random positions, players performed better in the check condition (medium complexity) than in less complex and more complex conditions. When the targets are visually simple (i.e., a king), performance is lower because knowledge of the stimuli structure does not guide attention to relevant locations. Since the target stimuli and distractors occupy one square each, discriminability is reduced, making detection difficult. When the target stimulus is a two-pieces interaction (i.e., a check), complexity increases. However, as the target spans a larger number of squares (3.9 on average) than randomly-distributed, individual distractors, it emerges as a single object. In the checkmate condition (maximal complexity), the effect is eliminated because the target pattern itself is of high complexity; since it cannot be retrieved from memory, it has to be computed in real time, hence the drop in performance. Strikingly, the novice and advanced players in our sample were affected by complexity in the same way, as indicated by the lack of significant interaction between expertise and complexity. Taken together, these findings suggest that complexity in visual signals can have a facilitator effect if the complexity of the target, but not of the full setting, is captured by memory chunks.

The trends analysis and our mathematical model are highly informative regarding how the structure of the environment modulates the perception of complex targets. While the trend in game positions is linear, random positions generated an inverted-U curve. More specifically, the mathematical model predicts that performance will peak for targets that encompass 10 squares and then will gradually decreases until it reaches 0% for 50-square targets; beyond this point, the model is not able to predict performance. With game positions, target detection is helped by the structure of the environment itself, while this cannot occur in random environments. It can be argued that the model is built upon a restricted number of points and as such might not be representative for high levels of complexity. Although we acknowledge the limits is predictability of the model, we reason that since human cognition is limited [22], [23], performance cannot increase indefinitely and so our model likely offers a first approximation of the inherent limits of pattern recognition.

A number of caveats should be noted in interpreting the present results. Although we view the influence of complexity results as very encouraging for the research of expertise acquisition, we are unable to generalize our findings to natural scenes. We encourage future research in the field to disentangle which components of the pop-out effect revealed in this paper also apply to visual search in natural scenes. A second caveat is that, although we enrolled advanced chess players and some experts, the results might not generalize to high levels of expertise. In this respect, future research should address at which level this pop out effect appears and at which level, if ever, it disappears. Another potential avenue for future research is to evaluate the relative weights of the visual and spatial components. In chess, visual and spatial factors are entangled so that our model does not differentiate between these two factors. Studies investigating the relative weights of spatial and visual information in loading the perceptual span could reveal which component draws more heavily on perceptual load. Such studies would prove useful in cognitive engineering. Also, in line with the measure of the limits of pattern recognition, it would be of high interest to use experts in other fields to contrast the limits found in the present research in chess expertise with those in related board games [24]. Combining estimates from different field of expertise would provide a more reliable estimate of the actual limit in perceptual span.

This study investigated the role of complexity in relation to expertise and the ecological validity of the stimuli in a visual search task. Increases in complexity led to different functional relationships for artificial and ecologically valid stimuli. A direct implication is that most of research in experimental psychology, which uses artificial stimuli, leads to conclusions that cannot be generalised to ecologically valid stimuli. While others have made this point (most notably [25]), it is particularly well illustrated in the current paper, as different mathematical functions (linear vs. quadratic) were observed in the game and random conditions. Thus, the difference was not only about the strength of the relationship between complexity and RTs. The difference was more drastic: the entire relationship was different for the two types of stimuli. This suggests that caution should be exercised when generalising from experiments using artificial stimuli with low ecological validity, which is the case with most experiments in cognitive psychology and cognitive neuroscience, to real-life stimuli.
